# Supplementary material for: Trace amine-associated receptors (TAARs)2-9 knockout mice exhibit reduced wakefulness and disrupted REM sleep
Source: Front Psychiatry. 2025 Jan 29;15:1467964. doi: 10.3389/fpsyt.2024.1467964 (PMC11814429; doi:10.3389/fpsyt.2024.1467964)
Supplement: Supplementary file 2 [file Table1.pdf]

Supplementary Table 1. Statistical Values of Supplementary Figure 1.

**Figure/Panel** (Time bin)	**Figure Title**	**Type of test**	***F* value**	***p* value**	**Significance**
**Figure S1. A**	**Wake (ZT1-3)**	2-way ANOVA (genotype)	F(1,108) = 1.252	0.2901	ns
**S1.B**	**NREM (ZT1-3)**	2-way ANOVA (genotype)	F(1,108) = 10.2	0.0018	**
**S1.C**	**REM (ZT1-3)**	2-way ANOVA (genotype)	F(1,108) = 15.54	0.0001	***
**S1.D**	**Wake (ZT4-6)**	2-way ANOVA (genotype)	F(1,108) = 12.09	0.0007	
**S1.E**	**NREM (ZT4-6)**	2-way ANOVA (genotype)	F(1,108) = 29.14	<0.0001	****
	β	Šídák's multiple comparisons test		0.0342	*
	Low γ	Šídák's multiple comparisons test		0.0161	*
	High γ	Šídák's multiple comparisons test		0.0079	**
**S1.F**	**REM (ZT4-6)**	2-way ANOVA (genotype)	F(1,108) = 21.07	<0.0001	****
	Low γ	Šídák's multiple comparisons test		0.0432	*
**S1.G**	**Wake (ZT7-9)**	2-way ANOVA (genotype)	F(1,108) = 6.437	0.0126	*
**S1.H**	**NREM (ZT7-9)**	2-way ANOVA (genotype)	F(1,108) = 34.36	<0.0001	****
	β	Šídák's multiple comparisons test		0.0255	*
	Low γ	Šídák's multiple comparisons test		0.0124	*
	High γ	Šídák's multiple comparisons test		0.0041	**
**S1.I**	**REM (ZT7-9)**	2-way ANOVA (genotype)	F(1,108) = 25.45	0.0005	
		Šídák's multiple comparisons test		0.0134	*
		Šídák's multiple comparisons test		0.0489	*
**S1.J**	**Wake (ZT10-12)**	2-way ANOVA (genotype)	F(1,108) = 18.65	<0.0001	****
**S1.K**	**NREM (ZT10-12)**	2-way ANOVA (genotype)	F(1,108) = 34.36	<0.0001	****
	β	Šídák's multiple comparisons test		0.0259	*
	Low γ	Šídák's multiple comparisons test		0.0262	*
	High γ	Šídák's multiple comparisons test		0.0099	**
**S1.L**	**REM (ZT10-12)**	2-way ANOVA (genotype)	F(1,108) = 28.17	<0.0001	****
	β	Šídák's multiple comparisons test		0.0458	*

	Low γ	Šídák's multiple comparisons test		0.0148	*
	High γ	Šídák's multiple comparisons test		0.0485	*

n.s: not sigificant, *, $p < 0.05$; **, $p < 0.01$; ***, $p < 0.005$; ****, $p < 0.0001$.
